# Single-Incision versus Multiport Robotic Myomectomy: A Propensity Score Matched Analysis of Surgical Outcomes and Surgical Tips

**DOI:** 10.3390/jcm10173957

**Published:** 2021-08-31

**Authors:** Sa-Ra Lee, Ju-Hee Kim, Young-Jae Lee, Shin-Wha Lee, Jeong-Yeol Park, Dae-Shik Suh, Dae-Yeon Kim, Sung-Hoon Kim, Yong-Man Kim, Young-Tak Kim

**Affiliations:** 1 Department of Obstetrics and Gynecology, Seoul Asan Medical Center, University of Ulsan College of Medicine, 88, Olympic-ro 43-gil, Songpa-gu, Seoul 05505, Korea; xjuheex@gmail.com (J.-H.K.); swhlee@amc.seoul.kr (S.-W.L.); catgut1-0@hanmail.net (J.-Y.P.); ddsuh@amc.seoul.kr (D.-S.S.); kdyog@amc.seoul.kr (D.-Y.K.); kimsung@amc.seoul.kr (S.-H.K.); ymkim@amc.seoul.kr (Y.-M.K.); ytkim@amc.seoul.kr (Y.-T.K.); 2 Department of Obstetrics and Gynecology, Gangneung Asan Hospital, University of Ulsan College of Medicine, 38, Bangdonggil, Sacheon-myeon, Gangneung-si 25440, Gangwon-do, Korea; lyjobgy@gmail.com

**Keywords:** single incision, minimally invasive surgery, myomectomy, robot-assisted laparoscopy

## Abstract

We aimed to compare the perioperative outcomes of single-incision robotic myomectomy (SIRM) and multiport robotic myomectomy (MPRM) and provide surgical tips. We retrospectively analyzed the medical records of 462 patients with symptomatic leiomyoma who underwent MPRM or SIRM between March 2019 and April 2021. Demographic characteristics and surgical outcomes, including the total operative time (OT), estimated blood loss (EBL), and surgical complication rate, were compared between the two groups. Patients in the SIRM group had lower a body mass index and rate of previous pelvic surgery and were younger than those in the MPRM group. The myoma type was not different between groups; however, the MPRM group had larger, and more myomas than the SIRM group. After propensity score matching, these variables were not significantly different between the groups. The total OT, EBL, difference in hemoglobin levels, transfusion rate, and postoperative fever were not different between the groups. No postoperative complications occurred in the SIRM group. In the MPRM group, one patient needed conversion to laparotomy, and two patients had postoperative complications (umbilical incisional hernia and acute kidney injury). In conclusion, both MPRM and SIRM are feasible and effective surgical options for symptomatic myomas with cosmetic benefits and minimal risk of laparotomy conversion.

## 1. Introduction

Uterine myomas are one of the most common benign gynecologic tumors in reproductive-aged women with a prevalence of up to 70% [1]. Myomectomy is an appropriate surgical option for patients who want to preserve their fertility [2,3]. The surgical methods for myomectomy have progressed from open abdominal myomectomy to minimally invasive surgery, such as laparoscopic myomectomy and robot-assisted laparoscopic myomectomy (RALM) [4,5,6,7]. Recent studies have shown that RALM may have advantages over laparoscopic myomectomy in terms of blood loss, postoperative transfusion, and length of hospital stay [8,9,10]. EndoWrist robotic surgical systems with three-dimensional vision enable multiport, single-port, and single-site RALM and are ergonomically excellent and user-friendly [8,11]. Therefore, RALM is advantageous because it allows the performance of multiple intracorporeal sutures easily and quickly. 

In terms of single-incision robotic surgery (SIRS), single-site robotic surgery using the da Vinci Xi^®^ or Si^®^ system (Intuitive Surgical, Sunnyvale, CA, USA) and single-port robotic surgery using the da Vinci SP^®^ system (Intuitive Surgical) are commonly performed [12]. Benign gynecologic surgeries, including hysterectomy, myomectomy, adnexal surgery, and sacrocolpopexy, as well as gynecologic oncologic surgeries, such as early endometrial cancer surgery, cervical cancer surgery, and pelvic lymphadenectomy, have been performed using SIRS systems [13,14,15,16,17,18]. SIRS is superior to multiport robotic surgery in terms of cosmetic outcomes [19] because SIRS requires only one intraumbilical skin incision, while multiport robotic surgery requires three, four, or five skin incisions. Some studies have reported that SIRS can provide better results with a shorter recovery time and less blood loss and surgical complications than multiport robotic surgery [20,21].

However, studies comparing single-incision robotic myomectomy (SIRM) and multiport robotic myomectomy (MPRM) are limited. Furthermore, myomectomy usually requires multiple intracorporeal sutures, and uterine myomas have diverse sizes and types and are often difficult to access laparoscopically, even when using robotic surgical systems. Owing to the technical difficulties of SIRM compared with MPRM, SIRM is performed in patients with small and few myomas and a low body-mass index (BMI) [22,23]. Nevertheless, after adjusting matched propensity scores (PSs), studies have shown that both MPRM and SIRM are safe procedures associated with low blood loss and minimal surgical complications.

Choosing between MPRM and SIRM is important not only for ensuring optimal surgical outcomes, including a shorter total operative time (OT), but also for reducing the risk of conversion to MPRM or laparotomy. However, more studies are needed to guide the choice between SIRM and MPRM in patients with diverse myomas. In this study, we aimed to compare perioperative outcomes, including the OT, between SIRM and MPRM. We also sought to evaluate the feasibility and safety of SIRM and describe some surgical tips for SIRM and MPRM. We hope that this study can provide gynecologic surgeons with insights that can guide their choice between SIRM and MPRM.

## 2. Materials and Methods

### 2.1. Study Design and Patients

This retrospective study included a total of 470 patients who underwent RALM at Seoul Asan Hospital between 1 March 2019 and 30 April 2021. We excluded MPRM using two skin incisions (*n* = 8), and a total of 462 patients were included in the final analysis. The type of surgery (SIRM or MPRM) was decided after a thorough discussion with the patient considering not only the surgical scar but also the size, type, and number of myomas. The preference of the patients and surgeons was also considered. This study was approved by the Asan Medical Center Institutional Review Board (approval no. 2021-0808), which waived the requirement for informed consent owing to the retrospective chart review design of the study.

We obtained the following data from each patient’s medical chart: age, BMI, parity, gravidity, medical history, history of previous pelvic surgery, and preoperative hemoglobin (Hb) level. The maximal diameter of the dominant myoma was measured on preoperative ultrasound images. Magnetic resonance imaging was further performed for accurate preoperative evaluation in the following situations: diagnosis of adenomyoma or malignancy, multiple myomas, or myoma adjacent to the endometrium. Myomas were categorized into submucosal (International Federation of Gynecology and Obstetrics (FIGO) types 0–2), intramural (FIGO types 3–4), subserosal and pedunculated subserosal (FIGO types 5–7), or intraligamentary, cervical, and parasitic (FIGO type 8) myomas [24].

The following data were collected to evaluate surgical and perioperative outcomes: concomitant surgeries, total OT (skin incision to closure), estimated blood loss (EBL), number of myomas removed, blood transfusion, weight of the removed myomas (as weighed during the pathologic examination), postoperative Hb level, length of hospital stay, postoperative fever within 48 h, and any complications during or after surgery. 

### 2.2. Surgical Procedures

All surgeries were performed under general anesthesia, and all patients underwent standard operative care. After anesthesia induction and draping of the patient, a RUMI Arch™ (CooperSurgical Inc., Trumbull, CT, USA) or ZUMI (CooperSurgical Inc., Trumbull, CT, USA) uterine manipulator was placed, and a Foley catheter was inserted into the bladder. MPRM was performed using the da Vinci Si^®^ or Xi^®^ system with central or side docking. A reduced-port method with a total of three skin incisions was used in all cases. A 2.5-cm intraumbilical incision was made, and one of the multichannel single ports, such as the Glove port (Nelis Meditech Inframed, Ojeong-gu, Bucheon City, Korea), Oneport (TEBA Corporation, Manan-gu, Anyang City, Korea), or S OnePort (ERAE SI, Hanam Technovalley U1 Center, Hanam City, Korea), was inserted for the camera and assistant laparoscopic instruments. Two 8 mm skin incisions were made on the left and right sides of the umbilical incision, 8–10 cm away from the umbilical incision, for two robotic trocars. Robotic instruments including a monopolar spatula bovie or monopolar scissors for the right robotic arm and fenestrated bipolar forceps for the left robotic arm were used for the uterine incision procedure. The right robotic arm was replaced with a Mega Suture Needle Driver^®^ during myometrial suturing.

For SIRM, single-site RALM was performed using the da Vinci Si^®^ or da Vinci Xi^®^ system, and single-port RALM was performed using the da Vinci SP^®^ system with central docking. An approximately 2.7-cm vertical incision was made in the umbilicus, and a multichannel single port, such as the Glove port (Nelis Meditech Inframed), the GelPoint (Applied Medical, Rancho Santa Margarita, CA, USA), Uni-Port (Dalim, Ojeoung-gu, Bucheon City, South Korea), or Lapsingle Vision (Sejong Medical, Paju City, Korea), was inserted. For single-port RALM, a metallic port specifically designed for the da Vinci SP^®^ system and 10 and 5 mm auxiliary ports were inserted into the preplaced multichannel single port. In the case of single-site RALM, semirigid robotic instruments including a monopolar hook bovie for the right robotic arm and fenestrated bipolar forceps for the left robotic arm were used for the uterine incision procedure. The right robotic arm was replaced with a Wristed Needle Driver^®^ (Intuitive Surgical) during the suturing procedures. In the case of single-port RALM, monopolar scissors for the right (3 o’clock) robotic arm, fenestrated bipolar forceps for the left (9 o’clock) robotic arm, and a large needle driver for the centrally positioned (6 o’clock) robotic arm were used. The arms of the monopolar scissors and large needle driver were interchanged for suturing procedures.

Myomas were retrieved from the uterus using robotic or laparoscopic tenaculum forceps in the usual manner or with the locking suture on myoma (LSOM) technique without the need for tenaculum forceps, as previously described [25,26]. The uterine myometrium and serosa were sutured layer by layer (in two or three layers) using a continuous running suture technique with absorbable barbed suture materials, including 1-0 Monofix PDO (Samyang, Daejeon, South Korea), 1-0 V-Loc™ suture (Covidien, Mansfield, MA, USA), or 1-0 Quill™ SRS bidirectional barbed suture (Angiotech Pharmaceuticals Inc., Vancouver, BC, Canada). The retrieved myomas were removed using knife morcellation within an Endopouch^®^ specimen retrieval bag (Ethicon, Johnson & Johnson, New Brunswick, NJ, USA) or an Endo-bag^®^ (Sejong Medical, Paju, Gyeonggi-do, Korea).

All patients received routine patient-controlled analgesia or nonsteroidal anti-inflammatory drugs administered intravenously three times a day until the second postoperative day. Thereafter, the intravenous patient-controlled analgesia was removed, or injected analgesics were changed to oral analgesics.

### 2.3. Statistical Analysis

To compare continuous variables between the MPRM and SIRM groups, we used Student’s t-test or Mann–Whitney U test. To compare the proportions of categorical variables between the two groups, we used a chi-square test or Fisher’s exact test. The data were normally distributed (*p* > 0.05, Kolmogorov–Smirnov test). We performed a statistical analysis of 15 single variables (myoma type, maximal myoma diameter, number of myomas removed, multiple myomas, number of large myomas >3 cm, weight of the removed myomas, LSOM use or not, total OT, EBL, preoperative and postoperative Hb levels, difference between preoperative and postoperative Hb levels, transfusion rate, length of hospital stay, and perioperative complications) to determine whether these variables were significantly different between the MPRM and SIRM groups. To minimize selection bias, 1:3 PS matching was performed with the single variables as dependent variables in the multiple regression analysis. The absolute standardized mean difference (SMD) was used to assess balance after weighting.

All computations were performed with R, a language and environment for statistical computation (R Foundation for Statistical Computing, Vienna, Austria) [27].

## 3. Results

### 3.1. Baseline Patient Characteristics

A total of 403 patients in the MPRM group and 59 patients in the SIRM group (59 cases: single site, *n* = 35; single port, *n* = 24) were included in this study. Parity, gravidity, history of pelvic surgery, and preoperative Hb level did not significantly differ between the MPRM and SIRM groups (*p* > 0.05). The SIRM group had a lower BMI (*p* = 0.0454) and younger age (*p* = 0.0002) than the MPRM group. The type of myoma was not different between groups; however, the mean maximal diameter of the dominant myoma was larger in the MPRM group (8.65 ± 2.76 cm) than in the SIRM group (7.6 ± 2.39 cm, *p* = 0.0059). The MPRM group (248 cases, 61.54%) had more cases of multiple myomas and more myomas >3 cm (median, 1; range, 1–11) than the SIRM group (multiple myomas: 26 cases, 44.07%; *p* = 0.0107; myomas >3 cm: median, 1; range, 0–4; *p* = 0.0018). The mean weight of the removed myomas was significantly higher in the MPRM group than in the SIRM group (301.86 ± 250.75 vs. 170.7 ± 146.8 g, *p* < 0.0001). The LSOM technique was used more frequently in the SIRM group than in the MPRM group (41 cases, 69.49% vs. 167 cases, 41.44%, *p* < 0.0001) (Table 1).

The adjusted 1:3 PS-matched data are shown in Table 2. A total of 148 patients in the MPRM group and 58 patients in the SIRM group were analyzed. A full nonparsimonious model was developed that included all variables listed in Table 1 and the interaction terms between variables. All absolute SMDs after matching were <0.25. After PS matching, BMI and age were not different between the two groups (*p* > 0.05). In addition, the maximal diameter of the dominant myoma (7.8 ± 2.5 cm), presence of multiple myomas (84 cases, 49.4%), number of myomas >3 cm (median, 1; range, 1–6), weight of the removed myomas (188.2 ± 128.0 g), and use of the LSOM technique (87 cases, 58.78%) in the MPRM group were not different from those in the SIRM group (*p* > 0.05). 

### 3.2. Surgical Outcomes

The total OT was longer in the MPRM group than in the SIRM group (146.9 ± 56.64 vs. 120.41 ± 52.99 min, *p* = 0.008) (Table 3). The MPRM group showed a higher EBL (317.4 ± 541.2 mL), a greater difference between the preoperative and postoperative Hb levels (3.02 ± 1.59 g/dL), and more cases of transfusion (73 cases, 18.11%) than the SIRM group, and these differences were significant (*p* < 0.05). After PS matching, the total OT, EBL, Hb level difference, and number of transfusion cases were not different between the two groups. The median length of hospital stay was 2 days (range, 2–22 days) in the MPRM group. Approximately 20% of patients showed postoperative fever within 48 h in both groups (83 cases (20.6%) vs. 11 cases (18.64%), *p* = 0.7282) (Table 3). In the MPRM group, there was one case of conversion to laparotomy and two cases of postoperative complications (umbilical incisional hernia and acute kidney injury).

## 4. Discussion

Both MPRM and SIRM were found to be feasible and effective surgical options for symptomatic myomas with cosmetic benefits and minimal risk of conversion to laparotomy.

SIRM was successful for myomas of any FIGO type, including subserosal, deep intramural, and submucosal myomas. SIRM and MPRM were successfully performed for myomas, with a median number of myomas of 1 (range, 1–7) and 2 (range, 1–34), respectively, indicating that patients with multiple myomas, rather than only patients with small numbers of myomas, can be candidates for RALM. SIRM and MPRM were effective for large myomas, with a mean myoma diameter of 7.6 ± 2.4 cm (range, 2–15 cm) and 8.6± 2.8 cm (range, 2–20 cm), respectively, suggesting that diverse-sized myomas including large-sized myomas can be treated with RALM.

SIRM has many advantages compared with single-incision laparoscopic myomectomy [28]. Single-incision laparoscopic myomectomy has unfavorable ergonomics and requires surgeons to have advanced skills to overcome crowding of instruments and limited depth perception [11,14]. Nevertheless, the type, size, and location of myomas can be obstacles, even for experienced laparoscopic surgeons, to perform multilayered myometrial sutures. Robotic surgical systems with EndoWrist technology (Intuitive Surgical) have nearly overcome these obstacles by allowing the articulation of instruments by up to 540° rotation and 45° angle for multiport, single-port, and single-site robotic instruments [22]. However, the limited range of motion with weak traction power for semirigid single-site robotic instruments and still-existing fighting between the instruments remain obstacles. Therefore, studies on single-incision laparoscopic myomectomy have been limited, and this may be the reason for the lack of randomized controlled trials comparing SIRM and single-incision laparoscopic myomectomy [29,30,31,32]. As shown in many studies on single-incision laparoscopic surgery versus SIRS for other gynecologic diseases, SIRS has the advantages of lower EBL and shorter hospital stay; however, opinions differ on whether robotic surgery is better than laparoscopic surgery in terms of reducing surgical complications and OT [15,33]. A meta-analysis of single-site RALM by Giovannopoulou et al. included four studies (a total of 267 patients) and demonstrated that single-site RALM was associated with an OT of up to 154 min and approximately 2% transfusion and postoperative complication rates regardless of the type and size of myomas [28]. In our study, the SIRM group (59 cases: single site, *n* = 35; single port, *n* = 24) had a short OT (120 min) and only three cases of transfusion. In addition, the interpretation is limited due to the small number of patients; however, the OT of single-site RALM (*n* = 35) was 104 min, and there were two cases of transfusion. Therefore, SIRM is considered a good surgical option for laparoscopic myomectomy. 

Nevertheless, single-site RALM allows a limited range of motion compared with the newly developed single-port RALM or MPRM. The limited EndoWrist rotation of the instrument tip, relatively long metal cannula, and semirigid instruments confer some limitations in intraoperative handling and strength when enucleating myomas or performing multilayered sutures [12,34]. For these reasons, many surgeons prefer MPRM, and thus far, only two observational studies and one systematic review have compared SIRM and MPRM [22,23,28]. Similar to the previous two observational studies, we observed that the SIRM group had fewer and smaller myomas that weighed less than those in the MPRM group. However, compared with the SIRM group in the previous studies, our SIRM group included the largest number of cases (*n* = 59), largest size of myomas (7.6 ± 2.4 cm), and heaviest removed myomas (170.6 ± 146.8 g). Furthermore, the total OT was not different between the SIRM and MPRM groups, and the total OT of the SIRM group in our study was the shortest (120.4 ± 53.0 min). The length of hospital stay was the shortest in Moawad et al.’s study [22], which included same-day discharge or overnight admission cases, and the longest in Choi et al.’s study [23]. However, this difference may be due to cultural and medical insurance regulations or routine post-operative care protocols on hospital stays for each country or hospital. 

Conversion to laparotomy was needed in one case in the MPRM group but in none in the SIRM group, similar to the reports in previous studies [22,23,28,30]. The patient who needed conversion was short in stature (height, 146 cm) and had multiple (>10) large myomas, which precluded successful MPRM because the range of motion was severely limited with the usual reduced-port method. For patients with a short stature and multiple large myomas, it is better to perform a high incision instead of an intraumbilical incision and additional skin incisions, with the use of advanced energy laparoscopic devices (which have a shorter working time than that of robotic hemostasis instruments for effective hemostasis) or the hybrid technique [34,35], to accomplish successful MPRM.

An umbilical incisional hernia occurred in one patient in the MPRM group. Studies on trocar-site hernia development after robotic gynecologic surgery are limited, and no other study has reported trocar-site herniation after a 2.5- to 2.7-cm incision at the umbilicus, as in our study. According to a previous study, 3 of 500 (0.6%) patients who underwent robotic gynecologic surgery developed incisional hernias, all of which occurred at 12 mm trocar sites and not at 8 mm trocar sites [36]. In our study, one or two additional 8 mm skin incisions were made at both sides of the umbilical incision for MPRM. No herniation through the 8 mm trocar sites occurred in all 411 patients in the MPRM group. Further studies are needed on the risk and prevention of postoperative umbilical incisional hernia formation related to larger incision sizes in SIRS than in conventional multiport laparoscopy using a 12 mm intraumbilical incision. Severe postoperative anemia requiring blood transfusion occurred in one patient in the MPRM group. The patient had multiple large myomas (a total of 10 leiomyomas of the intramural type with a maximum diameter of 10 cm). The preoperative Hb level was 14.6 g/dL. She received 4 pints of red blood cell transfusion during surgery with an EBL of 1000 mL. The total OT was 4 h. The immediate postoperative Hb level was 9.8 g/dL; however, intraoperative hypovolemia resulted in oliguria, disseminated intravascular coagulation, and acute kidney injury due to hypovolemic shock on the operation day. Her kidney function returned to normal after 5 months of hemodialysis.

Conversely, there were no cases of conversion to laparotomy, trocar-site herniation, or severe postoperative complications in the SIRM group. However, these results need to be interpreted with caution. Surgeons usually prefer MPRM over SIRM for patients with complicated myomas owing to expectations of adverse perioperative outcomes. Although our study used PS matching, it might not completely rule out the selection bias associated with surgeon preference.

In choosing between SIRM and MPRM for each patient, we propose the following considerations as surgical tips: First, although we used an intraumbilical incision for the robotic camera in all cases regardless of the size of the uterus, it would be better to use a high incision (i.e., an incision higher than the umbilical level) for a large uterus extending above the umbilical level. Second, when performing SIRM or MPRM for complicated cases that require the hybrid technique or the incorporation of procedures performed above the umbilical level, the time for docking and undocking the robotic system should be considered. The da Vinci Xi^®^ or SP^®^ system can provide easier docking and undocking than the Si^®^ or X system^®^. This can also be helpful for decreasing the total OT. Third, in cases of very large myomas that fill the entire abdomen, especially for subserosal-type myomas, the operation table can be tilted. This can aid in exposing the pedunculated site of the myoma because gravity can provide downward traction. Fourth, when choosing SIRM, caution should be taken with respect to the patient’s body dimensions, especially height. Patients with a short stature usually have a short distance between the umbilicus and the target organ (i.e., the uterus). In such cases, only an intraumbilical incision should be used for SIRM, not a high incision above the umbilicus, to ensure good cosmetic outcomes. This can be a large obstacle when performing SIRM because both the single-site and single-port systems require extra intrapelvic space to allow the instruments to work in full range of motion. Surgeons will experience more collisions among not only instruments but also trocars during SIRM using the single-site or single-port systems than during MPRM. Fifth, in cases of multiple myomas, our previously described LSOM technique is a simple and cost-effective surgical technique for RALM that can be readily performed by inexperienced surgeons [25,26]. The LSOM technique can reduce the OT by obviating the time for finding the removed myomas in the pelvic cavity [25]. In the analysis, according to the use of the LSOM technique, the LSOM group showed a shorter OT than the no-LSOM group, with a marginally significant difference (137.8 ± 61.15 min vs. 148.2 ± 52.67 min, *p* = 0.0529) (table not shown). Therefore, we considered LSOM as a matching variable. 

This study has several limitations. First, this was a retrospective study with a small sample size, especially for the SIRM group. Second, cases of both single-port and single-site RALM were included in the SIRM group. Although the single incision and the absence of available robotic tenaculum forceps are common aspects of these two types of RALM, the single-port robotic system has been developed to overcome the disadvantages of the single-site system; therefore, single-port RALM must be easier than single-site RALM for most gynecologic surgeons, especially with regard to multiple intracorporeal sutures. However, considering that no study has compared single-port and single-site RALM and only few studies have compared the single-incision RALM and MPRM, the results of this study may provide some information useful for the choice of RALM.

In conclusion, our study demonstrated that even patients with relatively large, multiple, and heavy myomas can be candidates for SIRM, with surgical outcomes (e.g., OT, length of hospital stay, and complications) comparable to those of MPRM. Both surgical methods have the benefit of minimizing the risk of cosmetic complications (e.g., umbilical incisional hernia) or conversion to laparotomy. Considering our surgical tips when choosing between SIRM and MPRM will decrease the failure of RALM, especially for less experienced surgeons.

## Figures and Tables

**Table 1:** Baseline and preoperative patient characteristics.

Characteristics	MPRM	SIRM	*p* Value	SMD
	(*n* = 403)	(*n* = 59)		
Age (years), mean ± SD	37.99 ± 5.77	34.97 ± 6.37	0.0002	−0.49886
BMI (kg/m^2^), mean ± SD	22.59 ± 3.67	21.58 ± 3.28	0.0454	−0.29103
Parity, median (range)	0 (0–4)	0 (0–3)	0.2233	−0.16775
Gravidity, median (range)	0 (0–7)	0 (0–6)	0.1106	−0.17344
Previous pelvic surgery			0.2259	−0.1845
0, *n* (%)	354 (87.84)	55 (93.22)		
1, *n* (%)	49 (12.16)	4 (6.78)		
Type of main myoma			0.0819	0.33831
Intramural, *n* (%)	293 (72.77)	35 (59.32)		
Submucosal, *n* (%)	12 (2.98)	4 (6.78)		
Subserosal, *n* (%)	80 (19.85)	15 (25.42)		
Others (intraligamentary, cervical), *n* (%)	18 (4.47)	5 (8.47)		
Maximal myoma diameter (cm), mean ± SD	8.65 ± 2.76	7.6 ± 2.39	0.0059	−0.4056
No. of myomas removed, median (range)	2 (1–34)	1 (1–7)	0.0065	−0.39706
Multiple myomas, *n* (%)	248 (61.54)	26 (44.07)	0.0107	−0.35545
No. of myomas >3 cm, median (range)	1 (1–11)	1 (0–4)	0.0018	−0.48179
Weight of the removed myomas (g), mean ± SD	301.86 ± 250.75	170.65 ± 146.79	<0.0001	−0.63865
Concomitant surgery, *n* (%)	48 (11.91)	10 (16.95)	0.2753	0.14376
Preoperative Hb level (g/dL), mean ± SD	12.52 ± 1.39	12.63 ± 1.19	0.564	0.08518
Method of retrieving myoma			<0.0001	0.58834
LSOM, *n* (%) No LSOM, *n* (%)	167 (41.44) 236 (58.56)	41 (69.49) 18 (30.51)		

Abbreviations: MPRM, multiport robotic myomectomy; SIRM, single-incision robotic myomectomy; SMD, standardized mean difference; SD, standard deviation; BMI, body mass index; Hb, hemoglobin; LSOM, locking suture on myoma.

**Table 2:** Baseline and preoperative patient characteristics after 1:3 propensity score matching.

Characteristics	MPRM	SIRM	*p* Value	SMD
	(*n* = 148)	(*n* = 58)		
Age (years), mean ± SD	35.91 ± 5.74	35.14 ± 6.28	0.3459	−0.12977
BMI (kg/m^2^), mean ± SD	21.64 ± 2.98	21.55 ± 3.30	0.8434	−0.03078
Parity, median (range)	0 (0–3)	0 (0–3)	0.3463	−0.09522
Gravidity, median (range)	0 (0–6)	0 (0–6)	0.1984	−0.11658
Previous pelvic surgery			0.7879	−0.046
0, *n* (%)	136 (91.89)	54 (93.1)		
1, *n* (%)	12 (8.11)	4 (6.9)		
Type of main myoma			0.7702	0.16239
Intramural, *n* (%)	98 (66.22)	35 (60.34)		
Submucosal, *n* (%)	9 (6.08)	3 (5.17)		
Subserosal, *n* (%)	33 (22.3)	15 (25.86)		
Others (intraligamentary, cervical), *n* (%)	8 (5.41)	5 (8.62)		
Maximal myoma diameter (cm), mean ± SD	7.75 ± 2.37	7.68 ± 2.33	0.8542	−0.02923
No. of myomas removed, median (range)	1 (1–30)	1 (1–7)	0.1501	−0.19359
Multiple myomas, *n* (%)	72 (48.65)	26 (44.83)	0.6302	−0.07664
No. of myomas >3 cm, median (range)	1 (1–5)	1 (0–4)	0.2486	−0.14847
Weight of the removed myomas (g), mean ± SD	195.89 ± 159.08	173.82 ± 146.38	0.3147	−0.14445
Concomitant surgery, *n* (%)	23 (12.54)	10 (17.24)	0.7699	0.04596
Preoperative Hb level (g/dL), mean ± SD	12.54 ± 1.27	12.63 ± 1.19	0.6596	0.07268
Method of retrieving myoma			0.1051	0.21316
LSOM, *n* (%) No LSOM, *n* (%)	87 (58.78) 61 (41.22)	40 (68.97) 18 (31.03)		

Abbreviations: MPRM, multiport robotic myomectomy; SIRM, single-incision robotic myomectomy; SMD, standardized mean difference; SD, standard deviation; BMI, body mass index; Hb, hemoglobin; LSOM, locking suture on myoma.

**Table 3:** Surgical outcomes before and after 1:3 propensity score matching.

	Total Data			In PS Matched Data		
	MPRM	SIRM		MPRM	SIRM	
	(*n* = 403)	(*n* = 59)	*p*-Value	(*n* = 148)	(*n* = 58)	*p*-Value
Total operative time (min), mean ± SD	146.9 ± 56.64	120.41 ± 52.99	0.0008	127.5 ± 61.68	121.4 ± 52.88	0.4461
Estimated blood loss (mL), mean ± SD	317.4 ± 541.2	192.97 ± 145.17	0.0005	303.1 ± 825.98	194.6 ± 145.91	0.1297
Postoperative Hb level (g/dL), mean ± SD	9.49 ± 1.64	9.99 ± 1.24	0.0066	9.93 ± 1.52	9.97 ± 1.23	0.8537
Difference in Hb level (g/dL), mean ± SD	−3.02 ± 1.59	−2.63 ± 1.14	0.022	−2.61 ± 1.26	−2.66 ± 1.13	0.8001
Transfusion, *n* (%)	73 (18.11)	3 (5.08)	0.0194	13 (8.78)	3 (5.17)	0.4316
Hospital stay (days), median (range)	2 (2–22)	2 (2–3)	<0.0001	2 (2–6)	2 (2–3)	<0.0001
Postoperative fever (within 48 h), *n* (%)	83 (20.6)	11 (18.64)	0.7282	24 (16.22)	11 (18.97)	0.6599

Abbreviations: PS, propensity score, MPRM, multiport robotic myomectomy; SIRM, single-incision robotic myomectomy; SD, standard deviation; Hb, hemoglobin.

## Data Availability

The excel data used to support the findings of this study were supplied by Sa Ra Lee under license, and requests for access to these data should be made to Sa Ra Lee, leesr@amc.seoul.kr.
